# Controllable Electrically Guided Nano-Al/MoO_3_ Energetic-Film Formation on a Semiconductor Bridge with High Reactivity and Combustion Performance

**DOI:** 10.3390/nano10050955

**Published:** 2020-05-18

**Authors:** Xiaogang Guo, Qi Sun, Taotao Liang, A. S. Giwa

**Affiliations:** 1 Chongqing Key Laboratory of Inorganic Special Functional Materials, College of Chemistry and Chemical Engineering, Yangtze Normal University, Chongqing 408100, China; guoxiaogang@yznu.edu.cn; 2 Material Corrosion and Protection Key Laboratory of Sichuan Province, College of Chemistry and Environmental Engineering, Institute of Functional Materials, Sichuan University of Science and Engineering, Zigong 643000, China; 3 College of Life Sciences, Chongqing Normal University, Chongqing 401331, China; 4 Faculty of Materials and Energy, Southwest University, Chongqing 400715, China; liangtaotao@email.swu.edu.cn; 5 State Key Joint Laboratory of Environment Simulation and Pollution Control, School of Environment, Tsinghua University, Beijing 100084, China; giwasegun@live.com

**Keywords:** nano-Al/MoO_3_ MIC, stable suspension, electrophoretic deposition, kinetics, micro initiator

## Abstract

Film-forming techniques and the control of heat release in micro-energetic chips or devices create challenges and bottlenecks for the utilization of energy. In this study, promising nano-Al/MoO_3_ metastable intermolecular composite (MIC) chips with an uniform distribution of particles were firstly designed via a convenient and high-efficiency electrophoretic deposition (EPD) technique at room temperature and under ambient pressure conditions. The mixture of isopropanol, polyethyleneimine, and benzoic acid proved to be an optimized dispersing agent for EPD. The kinetics of EPD for oxidants (Al) and reductants (MoO_3_) were systematically investigated, which contributed to adjusting the equivalence ratio of targeted energetic chips after changing the EPD dynamic behaviors of Al and MoO_3_ in suspension. In addition, the obtained nano-Al/MoO_3_ MIC energetic chips showed excellent heat-release performance with a high heat release of ca. 3340 J/g, and were successfully ignited with a dazzling flame recorded by a high-speed camera. Moreover, the fabrication method here is fully compatible with a micro-electromechanical system (MEMS), which suggests promising potential in designing and developing other MIC energetic chips or devices for micro-ignition/propulsion applications.

## 1. Introduction

In recent decades, increasing attention has been paid to energetic fuels with high energy density (e.g., metastable intermixed composites (MICs) or nanothermites). They can generate superior combustion performance, so they are widely used in high-efficiency propellants [1], welding auxiliary devices [2], pyrotechnics [3], and specialized igniters or energetic chips [4] for a variety of military purposes and civilian applications. Generally, MICs are regarded as excellent fuels, which generally consist of metal-fuel (e.g., Al, Mg) and oxidizers that include metal oxides (e.g., MoO_3_ [5], Fe_2_O_3_ [6], polyvinylidene fluoride [7], NiO [8], CuO [9], and iodates [10]). Compared to traditional micro-MICs, nano-structured MICs have been gradually verified as a promising candidate for highly reactive energetic materials or composites due to their higher heat-release properties, greater contact area between fuels and oxidizers, and faster detonation velocity [11,12,13]. Therefore, there is an emerging research area of interest in designing novel nano-structured MICs via facile techniques.

The Al/MoO_3_ MIC, as a desirable energetic system, has continuously aroused great interest, owing to its high heat of reaction (4698 J/g) and adiabatic flame temperature (3547 °C, higher than that of Al/Fe_2_O_3_, Al/CuO, etc.). Recently, different fabrication methods have been explored to prepare Al/MoO_3_ MICs or nanothermites for developing their exothermic performances, including the thermal co-evaporation method [14], magnetron sputtering [15,16], sonic wave-assisted physical mixing [17], and arrested reactive milling laser irradiation [18]. For example, M.R. Zachariah et al. designed Al/MoO_3_ MICs with different multilayer internal structures via the magnetron sputtering method on a semiconductor bridge as a promising micro-energy storage device, and analyzed the condensed state reaction process in the obtained nano-multilayered films [19]. E.L. Dreizin et al. reported low-temperature exothermic reactions in fully-dense Al/MoO_3_ nanocomposite powders fabricated by the arrested reactive milling technique [20]. In addition, Al/MoO_3_ MICs fabricated by the traditional mechanical mixing technique were shown to fuel a dramatic combustion exothermic process with a high burning rate of 100 ± 4 m/s and a high pressurization rate of 35 kPa/μs [21]. Nevertheless, it is relatively difficult to simultaneously meet the requirements of being low cost and easy to operate, with high-film-forming efficiency, using most reported methods. It is worth noting that using a portable electrophoresis, electrophoretic deposition (EPD) has technically demonstrated advantages in controllability of the composition and deposition efficiency for the target products from the charged micro/nanoparticles [22,23], or polymer molecules [24,25] in a stable suspension. The fabrication of Al/CuO and Al/NiO energetic films with uniform distribution was demonstrated by the K. T. Sullivan group [26] and the D. X. Zhang group [27], respectively. In our previous research work, the EPD technique was successfully used to prepare an Al/Bi_2_O_3_ MIC system [28]. Moreover, for practical application, it is essential to combine MICs with micro-electromechanical system (MEMS) technology [29] (i.e., so-called “nanoenergetics-on-a-chip” technology), constructing miniature energy-demanding devices with wide applications. However, there are few reports of the controlled design of MIC (e.g., Al/MoO_3_) chips via the EPD technique. 

Thus, for these reasons, a novel nano-Al/MoO_3_ MIC chip combined with Al/MoO_3_ nanolaminates and a typical micro-semiconductor bridge was firstly designed via the facile EPD method using isopropanol, polyethyleneimine, and benzoic acid as an optimized dispersion system. As a type of binary energetic chip, the composition of the nano-Al/MoO_3_ MIC can be affected mainly by the EPD dynamic behaviors of the fuel (Al) and oxidizer (MoO_3_). Thus, further exploration of this aspect was analyzed and verified theoretically. Finally, the heat-release properties and ignition test of the product were investigated.

## 2. Experimental Section

### 2.1. Reagents and Materials

Polyethyleneimine, PEG-2000, benzoic acid, and nano-Al powders (99.9%) were purchased from Aladdin Inc. Corporation. (Shanghai, China). Isopropyl alcohol was purchased from Kelong Industrial Inc., (Chengdu, China). The other reagents (including hydrogen peroxide and ethanol) from Sinopharm Chemical Reagent Co., Ltd. (Shanghai, China) were used as analytical grade purity without further purification. Deionized water (18.2 Ω) was used in all experiments.

### 2.2. Controllable Design of Nano-Al/MoO_3_ MIC

**Figure 1:**
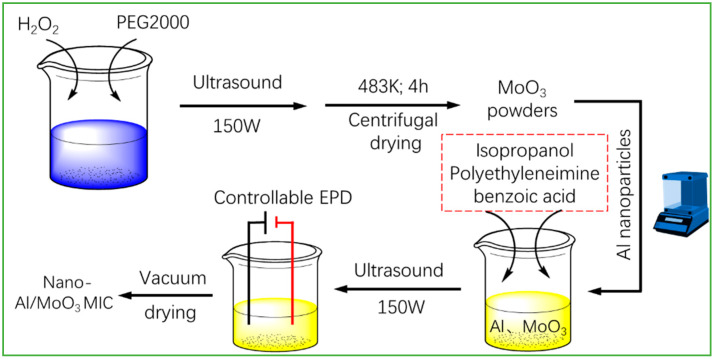
Schematic diagram of the facile fabrication of the nano-Al/MoO_3_ metastable intermolecular composite (MIC) chip.

In the fabrication of the nano-Al/MoO_3_ MIC, the EPD technique was exploited, and the corresponding detailed schematic diagram is displayed in Figure 1. Firstly, a classic one-step method was developed to prepare flake-like MoO_3_ powders. To be specific, 0.25 M Mo powders were added into 200 mL deionized water with trace PEG2000 marked as mixture *A*, then H_2_O_2_ (30 wt%) was dripped into mixture *A* slowly, until the yellow molybdenum peroxide sol appeared. After ultrasonic treatment for 0.25 h, the obtained sol was moved into a hydrothermal reactor at 110 ± 2 °C for 4 h, and the MoO_3_ powders were fabricated after repeated centrifugal cleaning at a rotation speed of 10,000 r/min and vacuum drying.

Then, the nano-Al and MoO_3_ powders with different mass ratios were added into the optimized dispersant of isopropanol, polyethyleneimine, and benzoic acid with a volume ratio of 50:1:1 to obtain a stable suspension after ultrasonic treatment for 20 min at 25 °C. During EPD, a micro-ignition bridge was the working electrode, and the copper sheet with the same area was used as the counter electrode; the detailed size of electrodes is shown in Figure S1 (Supporting Information). The distance of the two electrodes ranged from 0.4 to 2.4 cm. In addition, the EPD process was conducted under different field strengths. The EPD time ranged from 0 to 16 min. After EPD, the working electrode was removed from the suspension, and dried in an oven at 80 °C for 1.5 h. The nano-Al/MoO_3_ MIC chip was finally obtained after cooling to room temperature for the subsequent ignition experiments. The deposited efficiency (deposit weight per area (mg cm^−2^)) of the deposits was calculated by dividing the increased weight of the working electrode after EPD by the deposition area. Each experiment was repeated five times, and the average value of five parallel experiments was used as the final valid result.

### 2.3. Material Characterization

The morphology, element distribution, and crystalline structures of the nano-Al/MoO_3_ MIC were measured with a field emission scanning electron microscope (FESEM, JSM-7800F, Tokyo, Japan) equipped with energy dispersive X-ray spectroscopy (EDX), and X-ray diffractometer (XRD-6000, Shimadzu, Tokyo, Japan) with a scanning rate of 5°/min, respectively. Atomic absorption spectroscopy (AAS, 180-80, Exter Analytical, Tokyo, Japan) was used to determine the mass or mole ratio of Al and MoO_3_ in deposited energetic film. The heat-release (Q) of the energetic chip was analyzed by differential scanning calorimetry (DSC, STA449F3, NETZSCH, Berlin, Germany) measured in a temperature range from 25 to 1000 °C at a low heating rate of 10 K/min under a 99.999% argon flow. Ignition of the product was studied using home-made capacitor charge/discharge initiating equipment, and video recordings of the deflagration were recorded by a high-speed camera (Phantom v7.3, Vision Research, Inc., Wayne, NJ, USA) at an imaging speed of 10^4^ f/s.

## 3. Results and Discussion

### 3.1. EPD Dynamic Studies

**Figure 2:**
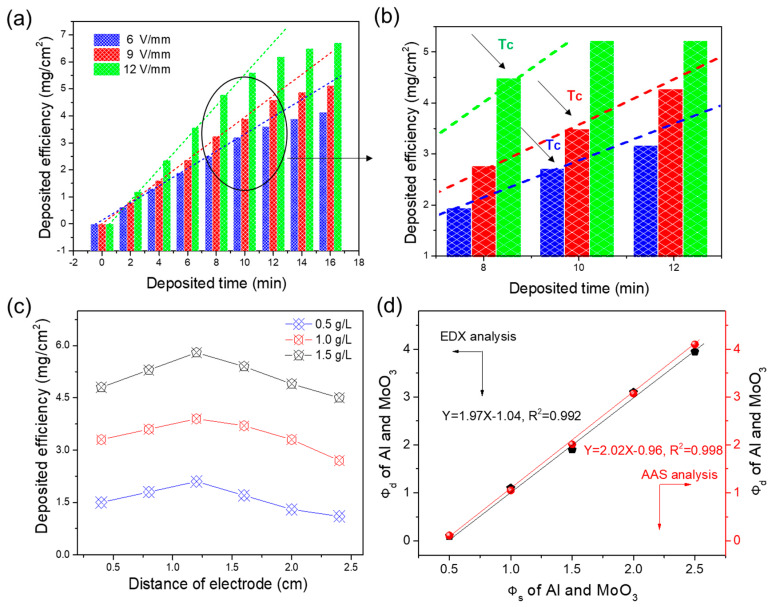
(**a**) Deposited efficiency (mg/cm^2^) of nano-Al/MoO_3_ MIC as functions of deposition time under different applied electric field strengths; (**b**) the local amplification image of the black circle in (**a**); (**c**) the relationship of deposited efficiency and the distance of two electrodes under different loading concentrations; and (**d**) Φ_d_ of Al and MoO_3_ in the Al/MoO_3_ MIC chip as a function of Φ_s_ of nano-Al and MoO_3_ particles in the starting suspension.

A successful EPD generally largely depends on various dispersing agents, and a large number of experimental studies have been carried out to compare the dispersion systems for EPD of specific sorts of particles [24,30,31]. After a large number of comparison attempts, the optimal dispersing agent of mixture of isopropanol, polyethyleneimine, and benzoic acid was used to fabricate the nano-Al/MoO_3_ MIC by EPD at normal tempearture and pressure. For verifying the EPD controllability, dynamic behaviours of particles in optimized suspension were studied in detail. Shown in Figure 2a is the deposited efficiency (mg/cm^2^) as a function of deposited time under applied electric field strengths ranging from 6 to 12 V/mm during EPD of Al/MoO_3_ MIC films. It was obviously observed that the deposition efficiency increased with the EPD time when the field strength was fixed at 6 V/mm, and a similar trend was also seen in a higher field strength of 9 or 12 V/mm. The higher field strength provides a higher EPD efficiency at a certain deposited time (e.g., 10 min). Moreover, the deposited efficiency increased linearly with deposited time increasing from 0 to T_c_ (the critical time between linear and non-linear EPD dynamic in the critical region (black circle)) in Figure 2a,b, which is consistent with the research of the Zhang group [32]. In addition, *T_c_* decreased with an increase of applied field strength; that is, *T_c_* for 6 V/mm was larger than *T_c_* for 9 and 12 V/mm, which was primarily due to the more severe precipitation and collision of particles under higher field strengths. Thus, the EPD process of Al/MoO_3_ MIC can be more precisely controlled in the linear control region (*t < T_c_*) in this study, which is due to the relatively complex relationship of deposited efficiency and EPD time in the nonlinear variation region for all field strengths. Furthermore, the effect of the distance of electrodes on the deposited efficiency of the nano-Al/MoO_3_ MIC is analyzed in Figure 2c. When the solid loading concentration, EPD time, and applied field strength were set at 0.5 g/L, 8 min, and 12 V (blue line), respectively, the deposited efficiency increased gradually with the distance of electrodes rising to 1.2 from 0.4 cm. It then decreased slowly, as the distance of electrodes continued to increase. This result is perhaps caused by the more violent disturbance of particles under a smaller distance of electrodes, and the higher degree of the settlement of particles under a longer distance of electrodes that leads to a lower EPD efficiency. Similar change trends were observed at higher field strengths (Figure 2c), which provides a valuable reference for realizing controllable EPD of different particles. 

In addition, the exothermicity of MICs is a key indicator that largely depends on the mass or mole ratio of fuel (e.g., Al) and oxidizer (e.g., MoO_3_). Generally, in MIC energetic reactions, the equivalence ratio (Φ) is defined as the actual ratio of fuel to oxidizer divided by the stoichiometric ratio of fuel to oxidizer in an energetic reaction, that is Φ = (F/O)_actual_/(F/O)_stoich_ [26]. For the codeposition process of the Al and MoO_3_ particles, the equivalence ratio in the starting suspension (Φ_s_) was adjusted accurately in weighed samples, and the equivalence ratio in the deposited product (Φ_d_) was determined by EDX and AAS techniques. Figure 2d displays the Φ_d_ of Al and MoO_3_ particles in the Al/MoO_3_ MIC chip as a function of Φ_s_ of nano-Al and MoO_3_ particles in the starting suspension. Clearly, it can be seen that Φ_d_ increased linearly with Φ_s_ by EDX and AAS analysis, and the fitted equations were similar (*Y* = 1.97*X* − 1.04, *R*^2^ = 992 for EDX analysis, and *Y* = 2.02*X* − 0.96, *R*^2^ = 998 for AAS analysis). Thus, Φ_d_ in the nano-Al/MoO_3_ MIC could be adjusted by changing Φ_s_ in suspension, which contributes to optimizing the proportion of components in product, and further developing the exothermic performance of the product.

### 3.2. Characteristics of Nano-Al/MoO_3_ MIC

**Figure 3:**
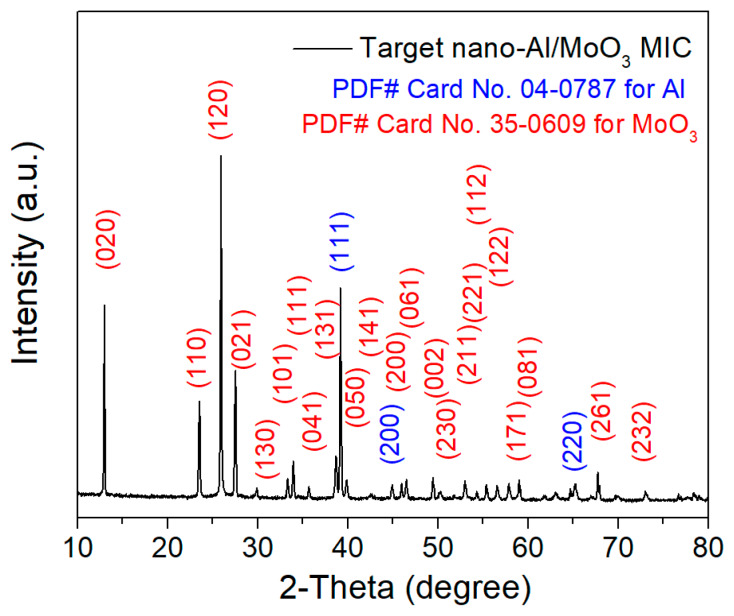
Typical X-ray diffractometer (XRD) pattern of the as-obtained nano-Al/MoO_3_ MIC.

XRD analysis was used to investigate the crystal structures of the nano-Al/MoO_3_ MIC in Figure 3. It can be clearly seen that two groups of distinct diffraction peaks are marked in good agreement with that of the standard spectra for pure Al (JCPDS card No. 04-0787; Fm-3m (225)) and MoO_3_ (JCPDS card No. 35-0609; Pb nm (62)) on the product, demonstrating the successful co-EPD of the Al and MoO_3_ particles. In addition, the fact that there are no other clear peaks of Al_2_O_3_ and Mo in Figure 3 indicates the high purity of the product, and that no thermite reactions took place during the EPD process.

**Figure 4:**
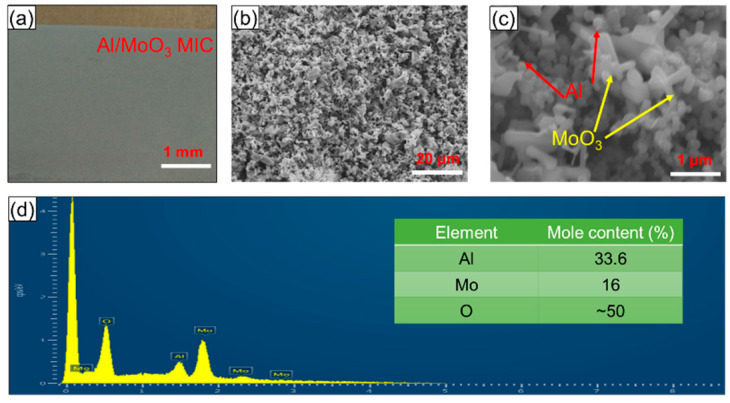
(**a**) Optical and (**b**,**c**) typical field emission scanning electron microscope (FESEM) images of the nano-Al/MoO_3_ MIC films prepared using electrophoretic deposition (EPD); (**d**) energy dispersive X-ray spectroscopy (EDX) spectrum of the product with an inserted table showing the mole content (%) of all elements.

The as-obtained nano-Al/MoO_3_ MIC films via EPD are displayed in Figure 4. Regions of large-scale local sags, crests, and separations are not seen optically in the target product surface (Figure 4a), which exhibits significant coating characteristics and uniformity. Clearly, in the FESEM image of product in Figure 4b, the nano-Al/MoO_3_ MIC appears to be uniformly distributed, without rare unmixed zones. The higher-resolution images in Figure 4c indicate that the nano-Al particles were scattered or distributed randomly in flake-like MoO_3_, which significantly helps to enlarge the contact areas of Al and MoO_3_, and shorten the mass transportation length (MSL) during the thermite exothermic reaction of nano-Al/MoO_3_ MIC. Moreover, there were numerous gaps among the particles (Figure 4b,c), contributing to providing a large number of heat-release channels or multiple spatial streams, and further improving the exothermic performance of the product [27]. Moreover, the elemental compositions in the nano-Al/MoO_3_ MIC were analyzed by the EDX technique, as shown in Figure 4d, where the EDX spectrum indicates that all expected elements of Al, Mo, and O existed in the energetic film surface, consistent with the results of the XRD analysis. It is worth noting that the mole ratio of Al, Mo, and O was close to 2:1:3 (0.336:0.16:0.50) (seen in Figure 4d and Table S1 in Supporting Information), and the corresponding reaction mole ratio of Al and MoO_3_ was close to 2:1, which contributed to realizing a sufficient aluminothermic reaction (2Al + MoO_3_ → Al_2_O_3_ + Mo + H_Heat−release_, ΔH = 4698 J/g) [17,33]. In addition, we conducted a comparative study of FESEM mapping and the corresponding results are similar in three random regions (Table S1), where the mole ratio of aluminum to nickel is close to 1:1, which indicates the uniform distribution of the product. Moreover, the percentage errors of the mole ratio of elements are approximate 5% in six random regions, according to both EDX and AAS analysis, further demonstrating the homogeneously mixed nano-Al/MoO_3_ MIC obtained by EPD.

### 3.3. Thermal Studies

**Figure 5:**
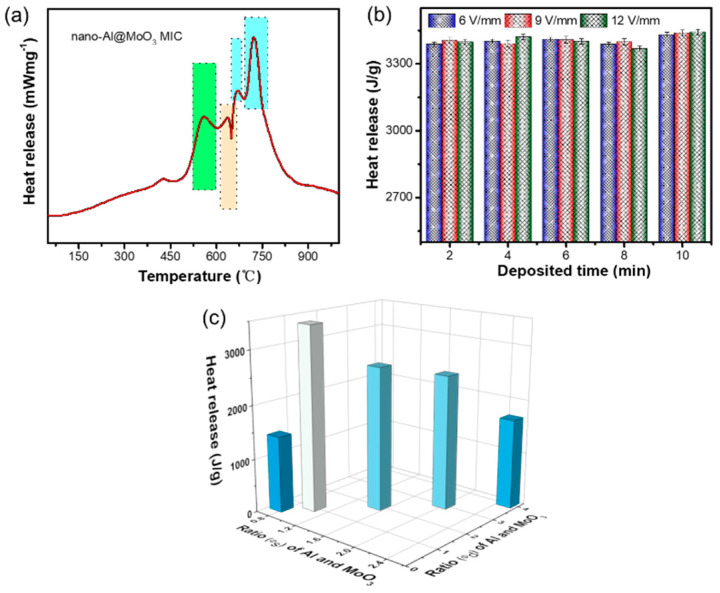
(**a**) Differential scanning calorimetry (DSC) curve of the obtained nano-Al/MoO_3_ MIC; (**b**) the relationship of heat release and deposited time under different applied electric field strengths; and (**c**) 3D histogram of heat release as a function of Φ_d_ and Φ_s_ of Al and MoO_3_ particles in the product.

Exploration of exothermic performance is essential to energetic materials, including MICs [34,35,36,37], and is shown in Figure 5 in detail. Figure 5a displays the DSC data measured from the nano-Al/MoO_3_ MIC with the Φ_d_ of ~1.0 and a low heating rate of 10 K/min. In addition to an unobservable exothermic peak at ~400 °C, probably due to the reaction between Al nano-particles with much smaller-sized MoO_3_ particles, there are several observable exothermic peaks in Figure 5a, where the exothermic peak (green rectangle area and yellow rectangle area) is mainly because of the reaction between Al particles with smaller-sized MoO_3_ particles [38], and the latter two exothermic peaks at 703.4 °C and 735.9 °C (blue rectangle area) are from the reaction of Al and bigger-sized MoO_3_ particles, which is consistent with the results from the Zhu group [15]. In addition, there was also an endothermic peak (Figure 5a, primrose yellow rectangle area) at ca. 660 °C, caused by the melt of metal-Al. After a fitting calculation, the value of the heat-release of the nano-Al/MoO_3_ MIC was as high as ~3340 J/g, which was >70% of the theoretical value, indicating the relatively sufficient thermite reaction. Furthermore, the effect of the deposited time on the heat-release of the product is analyzed in Figure 5b. There was a similar trend for different field strengths from 6 to 12 V/mm, that is, the output of heat was almost stable as the deposited time increased, showing the great controllability of EPD dynamic behaviors of Al or MoO_3_ particles in suspension. The heat-release values as functions of Φ_d_ and Φ_s_ of Al and MoO_3_ are clearly shown in the 3D histogram (Figure 5c). The heat-release values increased first and then gradually decreased with Φ_d_ of Al and MoO_3_, and were highly associated with Φ_s_ of Al and MoO_3_ in the starting suspension. The peak value of the heat release of the nano-Al/MoO_3_ MIC can be obtained when Φ_d_ of Al and MoO_3_ was close to 1.0.

**Figure 6:**
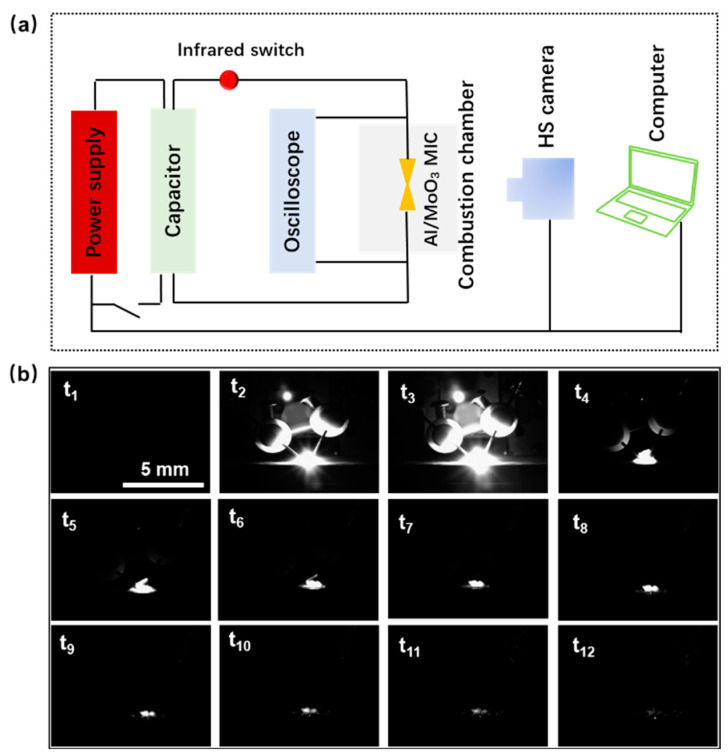
(**a**) Schematic of the ignition system for the micro nano-Al/MoO_3_ MIC chip initiator, and ignition process recorded by a high-speed camera; (**b**) series of still images taken from a typical ignition deflagration study of the nano-Al/MoO_3_ MIC fabricated by EPD process with Φ_d_ = 1.0; the time interval between images is 0.1 ms (*t_n_* − *t*_*n*−1_ = 0.1 ms, *n* ≥ 2).

The thermite reaction deflagration processes of an electric explosion for the nano-Al/MoO_3_ MIC chip were realized a self-regulating capacitor charge/discharge initiating device, and recorded synchronously by a high-speed camera. The detonation schematic diagram is displayed in Figure 6a. When the ignition circuit was switched on, the energetic target chip was quickly detonated with a dazzling blaze. The corresponding flame propagation images of the nano-Al/MoO_3_ MIC chip are observed in Figure 6b, where the interval time between adjoining images is 0.1 ms. The flame duration time of the nano-Al/MoO_3_ MIC chips was >1 ms, and loud sounds during the ignition test indicated that the thermite reactions were so intense that energy was released quickly [39,40,41,42]. In addition, the observed intense deflagration was in accordance with the DSC results, which provided a facile route to nano-MIC energetic chips for MEMS application. In addition, the heat-release performance of MIC chips can be optimized by building a theoretical bridge between the equivalence ratio of oxidant and reductant in starting suspension (Φ_s_) and target energetic films or initiators (Φ_d_).

## 4. Conclusions

In this study, a novel Al/MoO_3_ MIC chip initiator was firstly fabricated by a high-efficiency EPD technique in an optimized mixture dispersant of isopropanol, polyethyleneimine, and benzoic acid at normal temperature and pressure. The microstructures and chemical compositions of the product were demonstrated by FESEM, EDX, and XRD. The deposited energetic films exhibited even mixing between the oxidizer (Al) and reductant (MoO_3_), contributing to enhancing their exothermic performance. The EPD dynamic behaviors of nano-Al and MoO_3_ particles were studied, which can act as a theoretical bridge for connecting the Φ_s_ in starting suspension and Φ_d_ in energetic chips. DSC results showed the apparent exothermic peaks of nano-Al/MoO_3_ MIC chips, due to the thermite reaction between Al and MoO_3_, and the corresponding total heat-release was as high as ca. 3340 J/g when Φ_d_ of Al and MoO_3_ was close to 1.0. In addition, the Al/MoO_3_ MIC chip initiator can be successfully ignited with a typical capacitor charge/discharge ignition device, exhibiting outstanding detonation performance with a short burst time and a dazzling flame. In short, the design of the Al/MoO_3_ MIC chip initiator in this study will provide a universal approach for fabricating other thermite energetic chips with wide civilian and military applications, especially in micro-initiation or micro-propulsion systems.

## Supplementary Materials

The following are available online at https://www.mdpi.com/2079-4991/10/5/955/s1, Figure S1: The size specification of the working and counter electrodes used for EPD dynamic research and ignition test. All yellow rectangle zones are parts of electrodes without touching the optimized suspension., Table S1: Molar content results of different elements in products by EDX and AAS analysis in three random regions.
