# Supplementary material for: Controllable Electrically Guided Nano-Al/MoO3 Energetic-Film Formation on a Semiconductor Bridge with High Reactivity and Combustion Performance
Source: Nanomaterials (Basel). 2020 May 18;10(5):955. doi: 10.3390/nano10050955 (PMC7279552; doi:10.3390/nano10050955)
Supplement: Supplementary file 1 [file nanomaterials-10-00955-s001.pdf]

Supporting Information
for

Controllable electrically guided nano-Al/MoO_3 energetic-film formation on a semiconductor bridge with high reactivity and combustion performance

Xiaogang Guo [1,2], Qi Sun [3,*], Taotao Liang [4] and A. S. Giwa[5]

1 Chongqing Key Laboratory of Inorganic Special Functional Materials, College of Chemistry and Chemical Engineering, Yangtze Normal University, Chongqing 408100, China; guoxiaogang@yznu.edu.cn

2 Material Corrosion and Protection Key Laboratory of Sichuan Province, College of Chemistry and Environmental Engineering, Institute of Functional Materials, Sichuan University of Science and Engineering, Zigong 643000, China

3 College of Life Sciences, Chongqing Normal University, Chongqing 401331, China

4 Faculty of Materials and Energy, Southwest University, Chongqing 400715, China; liangtaotao@email.swu.edu.cn

5 State Key Joint Laboratory of Environment Simulation and Pollution Control, School of Environment, Tsinghua University, Beijing 100084, China; NasirA@tsinghuamail.edu.cn

* Correspondence: sunqi2017@cqnu.edu.cn

*Corresponding author E-mail: sunqi2017@cqnu.edu.cn (Q. Sun)

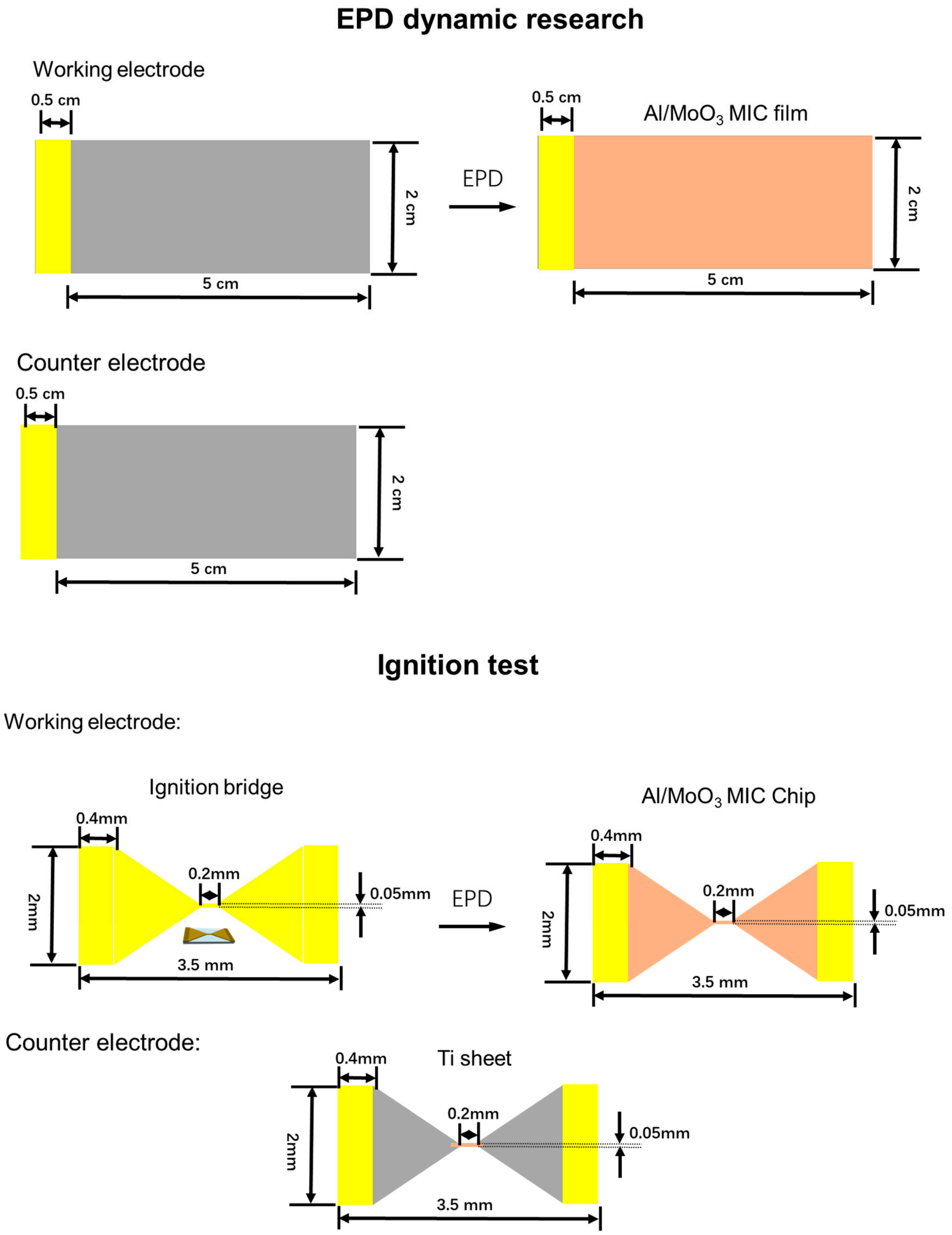

Figure S1. The size specification of the working and counter electrodes used for EPD dynamic research and ignition test. All yellow rectangle zones are parts of electrodes without touching the optimized suspension.

Table S1. Molar content results of different elements in products by EDX and AAS analysis in three random regions.

Elements	Molar content (%)							
	EDX analysis				AAS analysis			
	Region 1	Region 2	Region 3	Average value	Region 1	Region 2	Region 3	Average value
**Al**	~33.6%	~33.9%	~33.2%	~33.7%	~34.2%	~34.0%	~33.6%	~33.9%
**Mo**	~16%	~16.2%	~15.7%	~16%	~15.6%	~15.3%	~16.1%	~15.7%
**O**	~50%	~49%	~51%	~50%	~50.2%	~50.7%	~49.4%	~50.4%
	Al:Mo:O≈2:1:3				Al:Mo:O≈2:1:3			
